# Implementation Outcomes for Agitation Detection Technologies in People with Dementia: A Systematic Review

**DOI:** 10.3390/geriatrics10030070

**Published:** 2025-05-24

**Authors:** Nicolas Farina, Lorna Smith, Melissa Rajalingam, Sube Banerjee

**Affiliations:** 1 Faculty of Health, University of Plymouth, Plymouth PL6 8BX, UK; 2 Brighton and Sussex Medical School, Brighton BN1 9PX, UK; 3 Faculty of Medicine and Health Sciences, University of Nottingham, Nottingham NG7 2UH, UK

**Keywords:** agitation, detection, biosensing techniques, monitors, cameras, implementation, acceptability, feasibility studies

## Abstract

**Background**: Experiencing agitation can be particularly distressing for people with dementia and their caregivers. Using technologies to detect agitation can help monitor and intervene when agitation occurs, potentially reducing overall care and support needs. This systematic review aims to explore the implementation outcomes related to the use of agitation detection technologies in people with dementia. By adopting a taxonomy of implementation outcomes, this review seeks to provide insights valuable for the real-world adoption of such technologies for people with dementia. **Methods:** Searches were conducted in the following databases: SCOPUS, PubMed, PsychINFO, IEEEXplore, and CINAHL Plus. Included studies were required to have implemented, evaluated, or validated technology with the intention to detect agitation in people with dementia in real-time. **Results:** On 14 May 2024, 1697 records were identified, and 19 were included in the review. The median sample size was 10, and around two-thirds of the records (n = 12, 63%) used ‘multimodal’ technologies for detecting agitation. Over half of the records (n = 10, 53%) were reporting from two studies. Across technologies, there was evidence of acceptability and feasibility, though there was a general absence of primary data related to implementation outcomes. There were, however, a number of technical issues and limitations that affected the fidelity and appropriateness of the technology, albeit not unique to people with dementia. **Conclusions:** There is a need for more empirical data on this topic to maximise uptake and adoption. Future research needs to ensure that the voice of the person with dementia is integrated within the evaluation process.

## 1. Introduction

For people with dementia, cognitive impairment, including memory loss and attentional deficits, commonly disrupts their ability to perform vital daily tasks. These difficulties are amplified by the presence of neuropsychiatric symptoms such as agitation, depression, and anxiety [1]. Agitation, which is characterised by emotional distress, excessive motor activity, and/or aggression, ref. [2] is particularly distressing for people with dementia and carers [3,4].

In a study using electronic medical records, agitation was reported to affect 44.6% of people with dementia [5]. However, its prevalence depends on the outcome used and the sample population. There are a number of methods by which we can measure agitation through questionnaires and direct observation [6]. Recently, there have been efforts to detect agitation through the use of technology, to minimise observation bias and permit prolonged naturalistic monitoring. Such objectives are important, but we should also consider whether technologies can be used as a means of real-time detection of the earliest signs of agitation to permit early intervention and the prevention of harm [7].

A systematic review of sensor technology to monitor neuropsychiatric symptoms in dementia reported that nearly half (16/34) of the included studies represented proof-of-concept, acceptability, and/or feasibility testing [8]. However, we have little understanding of the barriers to implementing these types of sensing technologies in dementia. To help illustrate and understand the stages of digital technology development, the NIHR Older People and Frailty/Healthy Ageing Policy Research Unit suggest using Technology Readiness Levels (TRLs). Here, we see the assigned digital biomarkers collected through digital devices as being at the ‘ideas stage’ (TRL 0–3), and activity sensors to monitor distressed behaviours at the ‘prototype stage’ (TRL 4–5) [9].

Agitation detection technologies for people with dementia can be broadly split into *wearable sensors*, *computer vision,* and *multimodal sensors* [10]. Determining the validity of these technologies to detect agitation is essential. However, implementation outcomes are also paramount to understand implementation success, provide an indicator of the implementation process, and provide an intermediate outcome when considering efficacy [11]. A previous review on technologies used to detect agitation in dementia found that only three of the identified studies considered the technology’s acceptability [10], though the review did not synthesise these acceptability data or other implementation aspects.

There is the potential to learn about implementation outcomes by exploring the technology more broadly. Wearable sensors (e.g., actigraphy) and computer vision technologies (e.g., surveillance) have been adopted for a variety of purposes for people with dementia, with their acceptability and feasibility explored [12,13]. But it is important to consider the performance of technology, specifically in detecting agitation in people with dementia. For example, there are many potential triggers for agitation in dementia [14], and the introduction of technology might be one. Real-world and naturalistic settings also need to be explored, as the feasibility of implementing agitation detection technology could be affected by the setting, depending on who is supporting its implementation, or setting-based restrictions (e.g., privacy). Notably, the acceptability of a given technology is determined not only by ease of use but also by the perceived usefulness of the technology, as set out in the Technology Acceptance Model [15]. Agitation detection monitoring might be seen to be more acceptable if it is used as a mechanism for intervention.

Previous reviews [10,16] have touched on implementation outcomes for agitation detection technologies for people with dementia. Yet, these reviews do not systematically define, synthesise, or extract implementation outcomes. Understanding whether there are recurring barriers to implementation and what they look like will allow future technology developers to better meet the needs of people living with dementia and agitation. In addition, we seek to describe the extent to which early-stage implementation outcomes [11] are reported to identify whether there is scope to improve implementation research within this area. In this review, we ultimately provide recommendations to consider for future research seeking to develop agitation detection technologies for people with dementia.

## 2. Materials and Methods

The systematic review was guided by the Joanna Briggs Institute (JBI) Manual for Evidence Synthesis [17], followed a previously published protocol registered at Protocols.io [18], and conformed to the Preferred Reporting Items for Systematic reviews and Meta-Analyses (PRISMA) checklist [19]. See Appendix A for checklist.

### 2.1. Research Questions

1. What are the reported implementation outcomes related to real-time agitation detection technology in people with dementia?
2. What evidence is there to show that real-time agitation detection technologies can be implemented with people with dementia?

### 2.2. Defining Early-Stage Implementation Outcomes

We adopted the taxonomy of implementation outcomes reported by Proctor and colleagues, selecting outcomes that are salient to the early implementation stage [11]. These outcomes and definitions are provided in Table 1.

### 2.3. Search Strategy

An extensive search of five databases (PubMed, SCOPUS, PsycINFO, CINHAL Plus, and IEEXPLORE) was conducted. The search terms were devised so that we could capture the population (e.g., people with dementia), the technology (e.g., wearables), the function of the technology (e.g., detection), and the symptoms (e.g., agitation) (see Appendix B for example search syntax). No search terms were used to limit by outcome, because during pre-testing, this led to an increase in false negatives.

Inclusion criteria:

- Studies that have implemented, evaluated, or validated technology with the intention of detecting agitation in people with dementia in real-time. The study could use technology to detect agitation for monitoring purposes only or alongside agitation reduction interventions. These included technologies that aimed to achieve real-time agitation detection now or had plans to do so at a later stage of the study.
- People with dementia were required to be the target population in receipt of the agitation detection technology. There was no restriction on the subtype of dementia, the severity, or the residential status of the participant.
- Studies with one or more implementation outcomes related to the agitation detection technology. Studies were not required to frame the research as implementation science or have aims pertinent to these outcomes.
- Studies could report outcomes qualitatively or quantitatively.
- Written in English language.

Exclusion criteria:

- Studies that used agitation detection technologies as a secondary outcome as part of a broader research question (e.g., embedded within cohort studies).
- Studies that exclusively reported on the secondary analysis of data from technologies.
- Studies that had designed the technology for use in people with dementia but had not tested it in this population.
- Lab-based studies.
- Non-primary data studies (e.g., reviews, protocols, and editorials).

Studies that partially met the criteria (e.g., subset of sample had dementia and subset received agitation detection technology) were excluded where data could not be meaningfully extracted for this group.

### 2.4. Selection Process

Individual hits were downloaded from databases and merged into a single platform (Zotero), where deduplication occurred. The deduplicated hits were then uploaded onto ASReview [20] to allow for semi-automated screening of the title and abstract by a single reviewer (LS). Screening was informed by a decision tree. The reviewers (LS and NF) prompted ASReview with examples of eligible and ineligible reports (three of each). Based on the reviewers’ responses, ASReview uses machine learning algorithms to present the reviewer (LS) with records that have higher probability of being relevant. Screening stopped once a minimum of 10% of titles/abstracts were screened, and then following 50 consecutive screen negatives. The full texts of the shortlisted reports were reviewed independently (LS and NF), with disagreements discussed and consensus reached.

### 2.5. Data Extraction and Items

Data were extracted independently by one reviewer (LS), then verified by a second reviewer (MR). These data included descriptive information on the publication (inc. author and date of publication), the study (inc. type of funding and country of study), the agitation technology used (inc. type of sensor and duration of detection), and the population and study setting. Data on implementation outcomes were also extracted, including whether the report cited implementation outcomes or concepts in the report’s aims, and whether the reported outcomes were primary (from the consumer, consumer-by-proxy/carer, or the organisation/setting-level) or secondary (as researcher commentary). Data relating to implementation outcomes could be reported qualitatively (as themes, subthemes, and/or quotes) or quantitatively (descriptive data from questionnaires or scales with face validity) (see Table 1 for the types of data extracted). We extended the scope of the data extraction to include researcher commentary in the discussion sections of reports.

### 2.6. Critical Appraisal

To critically appraise the quality of the reports identified through this review, we adopted the Quality Assessment with Diverse Studies (QuADS) Criteria [21], as this tool can handle studies with a heterogeneous study design. To our knowledge, there are no widely used and validated tools specifically to appraise implementation science studies. As such, appraisal scores were used as a generic indicator of study quality, without any commentary on implementation sciences. The critical appraisal was completed independently by two reviewers (LS and MR), and any disagreement was resolved through discussion amongst the research team. We used the critical appraisal scores to contextualise the literature.

### 2.7. Data Synthesis

We provided a broad overview of included studies, including study and technology characteristics, alongside the critical appraisal. Findings were then synthesised narratively and split into technology types (i.e., video, wearables, and multimodal), as described in a previous review [10]. Within each technology type, each of the implementation outcomes was described where available. For multimodal technology instances where an implementation outcome specifically relates to a specific element of technology (e.g., wearables), these were further synthesised within the specific technology.

### 2.8. Meta-Bias

No formal analysis was employed to address meta-bias. However, this is discussed narratively below. Attempts to include non-peer-reviewed articles were used to minimise sources of publication bias.

### 2.9. Confidence in Cumulative Evidence

No formal assessment of confidence in cumulative evidence was used due to the heterogeneous nature of the studies and study outcomes (e.g., qualitative and quantitative).

### 2.10. Reporting Bias

Missing data were not actively sorted by authors. We narratively described and reflected upon gaps in data.

## 3. Results

### 3.1. Overview of Studies

**Figure 1:**
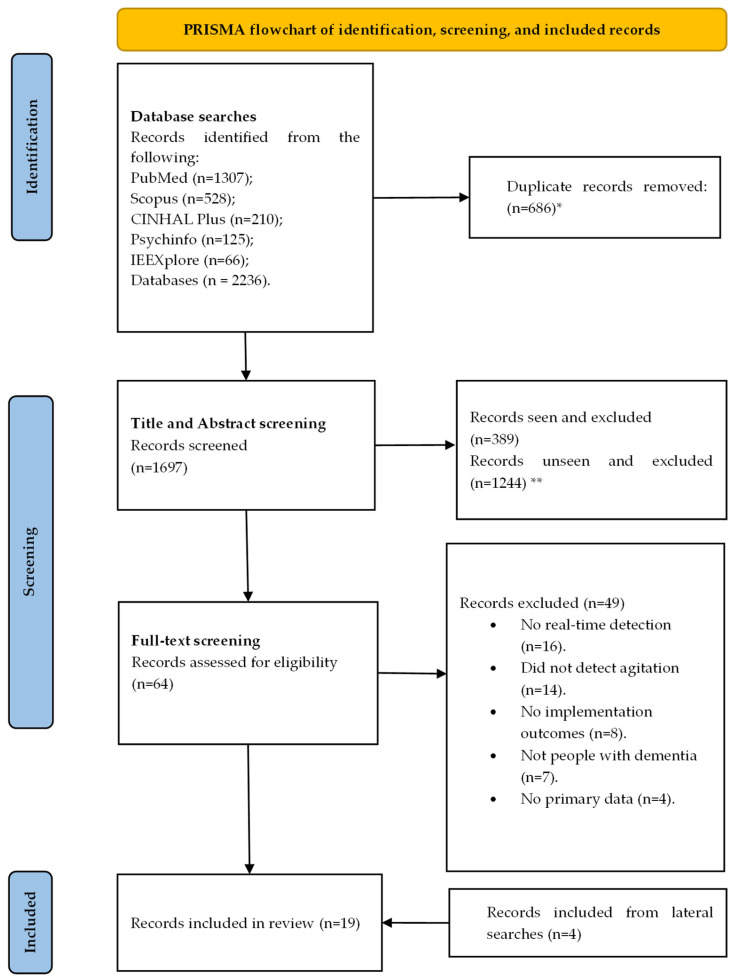
PRISMA flow diagram. * n = 539 ‘duplicate records identified’—some of the same duplicated reports were identified more than twice. ** using AS Review, reviewed n = 453 (10% + 50 irrelevant records since last relevant) of included records.

On 14 May 2024, the searches identified 1697 records, which, following deduplication, went through to the screening stage. Titles and abstracts were independently screened with the support of ASReview. Based on the a priori criteria, 453 abstracts were screened, of which 64 were included for the full-text review stage. Full-text review happened independently and in duplicate, with 15 records meeting the criteria for inclusion. Agreement was good between reviewers (K = 0.81). Following the review of reference lists and chasing citations, an additional four records were identified. So, there were 19 records included in this review; see Figure 1.

### 3.2. Study Characteristics

The records had relatively small sample sizes of people with dementia, ranging from several n-of-1 studies (n = 4, 21%) to 20 participants [22]. The median sample size was 10. Dementia type and demographic data were not frequently reported, nor were the indices of baseline cognitive impairment or agitation. All studies were from North America (n = 11, 58% USA; n = 5, 26% Canada) and Europe (Germany n = 1, 5%; France n = 1, 5%; Belgium n = 1, 5%).

Around two-thirds of the records (n = 12, 63%) used ‘multimodal’ technologies for detecting agitation. These could be a combination of wearable (including actigraphy), ambient sensors, pressure mats, video cameras, and acoustic sensors (including microphones). Four (21%) records used exclusively wearable technology, including bracelet sensors to monitor actigraphy (movement) and/or ambience (light, air pressure, loudness) [23,24,25,26]. Two (10%) used an ambient sensor to detect movement and activity, including infrared [27,28], and one (5%) used camera-based (computer vision) technology [29].

### 3.3. Overview of Technologies to Detect Agitation

Over half of the records (n = 10, 53%) were from two studies: BESI (including sub-study CANIS; n = 5, 26%) and DAAD (n = 5, 26%). Due to the varying sample sizes and stages of reporting, we decided to synthesise the results of the reports separately, rather than group records together if belonging to the same study. Over half of the reports used devices designed to collect data continuously across the duration of their deployment (n = 11, 58%). The deployment periods varied between <1 day and 138 days, with an average of 45 days. The majority of records (N = 12; 63%) were from non-community settings, including hospitals (N = 7, 37%) and residential care homes (N = 5, 26%); see Table 2 for further information.

### 3.4. Critical Appraisal

We judged the average study quality to be 70% (calculated as a percentage of the total possible score) on the QUADS, ranging from 26% [27] to 92% [31]. Across the studies, recruitment data were the domain that scored the lowest due to many studies not providing details about the number of people approached [36]. The other domain that we judged to be particularly poor performing was the lack of evidence that the stakeholders had been considered in the research design and conduct. In many instances, stakeholder involvement or considerations were briefly stated; see Appendix C for further information.

### 3.5. Implementation Outcomes

Only a quarter of the included studies referred to implementation outcomes in the aims reported (n = 5, 26%). These could be broad, such as aiming to address ‘implementation challenges’ or the ‘feasibility of device’, or more specific in terms of how the technology might be accepted or considered appropriate for its users, namely, people with dementia, caregivers, or healthcare staff. Some implementation outcomes were specific to the study (e.g., the cost of the study) rather than the technology itself. For the most part, the implementation outcomes were reported through researcher commentary. Some commentaries were derived from primary data reported in the records, though others appear to be derived from unreported data or researcher anecdotes. No records reported on adoption outcomes; see Table 3.

### 3.6. Multimodal Sensors

#### 3.6.1. Acceptability

In the limited evidence available, multimodal systems were generally well accepted by people with dementia. Rose and colleagues noted that the non-invasive nature of the technology used had not ‘bothered the patients’ [38]. Another study inferred that, as the agitation detection was performed using ambient sensors and did not interfere with the participants’ activities, it would be deemed as acceptable for people with dementia [32], though there were no data to support this. Interestingly, the agitation detection technology was less well received by carers, particularly in those who were not as technologically literate. Carers were concerned about doing things correctly, which added burden [38]. This was not universal across different systems, as the BESI system was seen as being easy to use by carers: ‘They were not a bother to me. I was actually called to fix things myself’ [31], and were ‘easy to use and unobtrusive’ [37].

For staff members, multimodal systems did not add to staff burden, and there were no complaints from nursing staff in relation to interfering with their work [32]. There were some concerns raised about the aesthetics of wall sensors from the CANIS study [31], with 60% (n = 6) of the carers finding the sensors to be an ‘aesthetic problem’. Quotes from the carers in this study related how some found the wall sensors ‘intrusive’, and found the rudimentary look of the sensors made them more noticeable and liable to getting knocked off the wall. There were general ‘privacy concerns’ flagged in one study [34]. Although the authors did not clearly highlight who and what this was in relation to, it is likely linked to the use of cameras in agitation detection.

There were instances where agitation detection resulted in an intervention, for example, in the BESI system, carers were notified in instances of agitation. When asked how the notifications affected them, 70% (n = 7) of carers responded that it was ‘no problem’ or ‘it didn’t’ and that it was not intrusive. One carer reported that their experience was generally negative (‘after a while, it was irritating since it was too late’). Overall, 70% (n = 7) of respondents in the CANIS study alluded to positive feelings about the automated intervention [31]. Homdee and colleagues also noted that notifications could empower carers to reengage with the person with dementia, even if notifications were false positives [37], thus indicating acceptability.

#### 3.6.2. Adoption

No records included outcomes related to the adoption of the technology.

#### 3.6.3. Appropriateness

For technology that uses mounted sensors, agitation detection could not occur outside of the home [31], thus limiting its appropriateness for people with dementia who spend time outside the home. The DAAD study noted that ‘due to high skewness in the data, the algorithms may still suffer from a high false alarm rate…’ [22], reflecting the (in)appropriateness of the algorithms used.

#### 3.6.4. Feasibility

There is mixed evidence about whether the multimodal systems were feasible. There were sometimes issues with ensuring that there was sufficient staffing to ensure data were being collected [34], and the ability of staff members to follow the protocols to ensure successful data collection [35,40]. In addition, there were also challenges with ensuring the multimodal system was appropriately installed (e.g., by a qualified electrician, with required supplies) and did not interfere with the daily running of the study setting (e.g., early morning) [40]. Ye and colleagues noted how the installation took 5 months as it had to be performed in early mornings when patients were asleep and before staff started work.

Due to the reliance on the internet by some technologies, the ‘digital capacity’ of the setting where the technology was being implemented could present challenges. This included hospitals, where network stability, signal strength, and range could present limitations [40], and rural areas, where internet access and speed are limited [33]. For systems that included wearables, there was a specific issue with battery life [26] (see Wearables, Feasibility). Outside technical issues, it was noted that using a single model to detect or predict agitation may not be possible due to the different triggers and symptoms of agitation experienced between people with dementia [33].

#### 3.6.5. Fidelity

While there was one report that data collection occurred ‘without technical difficulty’ [32], many multimodal systems evaluations demonstrated that the technology was not monitoring and/or collecting agitation data all the time. In some instances, this remained relatively low, with 2% of missing data [33], though in one study, there was missing data 52% of the time [32]. It was not always clear why there were gaps in data collection, whether it was a technological issue and/or a validity issue. For example, in one study, only 54% of reported agitation events were detected by the technology [37], though the cause of this was not discussed. Missing data were also attributed to Wi-Fi or connectivity issues [32,35] or imprecise sensor readings and motion artifacts [39]. In one study, there was a report of a system failure in one participant where no data were collected [36].

#### 3.6.6. Implementation Costs

Not reported.

### 3.7. Wearables

Four studies reported implementation outcomes exclusively related to wearable technologies [23,24,25,26]. However, within multimodal devices, there were instances where implementation outcomes were reported or discussed separately for wearables [30,32,33,35].

#### 3.7.1. Acceptability

Staff reported that there was no general rejection of wearable bracelets amongst participants, and that, in fact, there was positivity surrounding the devices, seeing them as something special [26]. There appears to be evidence of the perception that wearables would not be suitable, with one caregiver believing wearables would be irritating for the person with dementia and therefore declined participation [36].

Bankole and colleagues used the critical incident technique to assess the usability of their body sensor network [24]. There were 76 critical incidents across all observations of participants. The majority of incidents were related to participants adjusting the nodes (n > 30) and participants removing nodes (n > 10), potentially indicating issues with participant comfort or frustration with the wearables. The authors noted that this was expected as people with dementia ‘have a tendency to “fiddle” with things within easy reach’. The notion that participants would interact and remove wearable devices is reported elsewhere, with 32 instances of devices being taken off by participants [26]. Bankole and colleagues noted that from unstructured interviews, staff and family members showed strong resistance to a waist-mounted device but encountered little to no issues with wrist- and ankle-mounted devices, which patients could easily mistake for a wristwatch or the ankle security alert sensors commonly used in nursing homes. Regarding the BESI system [23], there was a general commentary that residents did not have any physical- or privacy-related interruptions for the duration of wearing a smart watch (30 days), potentially indicating the wearable’s acceptability.

#### 3.7.2. Adoption

Not reported.

#### 3.7.3. Fidelity

The fidelity of people with dementia wearing the technology was generally good. For example, in one study, an Actiwatch was only detected as taken off 2% of the time [32]; others reported that wearables ‘did not miss any physical agitation episodes’ [33]. It was noted in one study that when participants were most agitated that they were more likely to remove the wearable; however, outside of these times, participants were not always aware of them [35]. There were underlying tech issues that meant that wearables were not delivered as planned. Sometimes this was because the wearables malfunctioned [31] or the status screen of the wearable was unintelligible or misleading [26]. In other instances, tech issues meant that data were not sent or received from the wearables. For example, the wearable collected about 60% to 90% of expected data [30]. Tiepel and colleagues noted that issues of non-recording could be attributed to human error, in which staff (i.e., nurses) forgot to start recording (32 instances) or attach the wearables incorrectly [26]. Bankole and colleagues noted issues with wireless problems (n < 10) and with laptop batteries (n < 5) when data were being streamed to a Bluetooth transceiver [24]. Tiepel and colleagues reported 32 occurrences where no recording was made for unreported reasons [26].

#### 3.7.4. Feasibility

Feasibility outcomes were often not reported, though it does appear that some wearables were not feasible, with compliance fluctuating in up to 4 of the 17 participants over the day in one study [26]. There were also instances where the batteries of the bracelets ran out of power, affecting the wearable’s activity [26].

#### 3.7.5. Appropriateness

Regarding wearable audio sensors, Nesbitt and colleagues commented that they had difficulty in differentiating between participants’ voices and the voices of others as they captured ambient sounds, noting that the use of voice sensors might be better suited to home rather than crowded environments [25]. In one study, the wearables required wireless linkage to a fixed system, meaning leaving the house resulted in gaps in data [30].

For wearable bracelets, one study used a watch-like device designed to display the time at the push of a button. However, as some participants were no longer able to push the button, the suggestion of having the time permanently displayed was suggested to be more useful [26]. In addition, the design of this device included a clasp that was easy to open, meaning participants could easily take off the wearable sensor.

#### 3.7.6. Implementation Costs

Not reported.

### 3.8. Other Ambient Sensors

Two studies included in the review exclusively explored ambient sensor technologies to detect agitation. One study used radio waves to infer patient movement, spatial location, and activity [28], while the other used passive infrared sensors or ‘passive teleassistance’ to detect changes in movement [27]. Nine of the studies using multimodal systems adopted other ambient sensors as part of the agitation detection, including in-home environmental sensors, pressure mats, and acoustic sensors. Two reported on implementation outcomes relating specifically to the other ambient sensor technology [30,32].

#### 3.8.1. Acceptability

No primary reports of acceptability outcomes were included in either study, although Vahia and colleagues noted that no complaints or adverse events were reported by staff. The authors also noted that there were no attempts to ‘…move, dislodge, or damage the device’ [28].

#### 3.8.2. Adoption

Not reported.

#### 3.8.3. Appropriateness

Regarding appropriateness, Banerjee and colleagues noted how the infrared sensors used in their study were unable to detect patient activity outside of the room where the sensors were installed [27].

#### 3.8.4. Fidelity

Vahia and colleagues reported on the fidelity outcome of their ambient sensors [28], as the sensors were able to transmit 96.2% of collected data. The remaining data were lost due to Wi-Fi-related outages. As part of a multimodal design, infrared motion detection sensors were used in one study, reporting no technical difficulty for the duration of deployment (138 days) [32]. Alam and colleagues describe that data loss from environmental sensors in the BESI system was reduced to an average of 1% during its development [30].

#### 3.8.5. Feasibility

Not reported.

#### 3.8.6. Implementation Costs

Not reported.

### 3.9. Camera-Based

The DAAD study used camera-based technology as part of the multimodal agitation detection monitoring. Although implementation outcomes related to the camera-based technology were touched on in several of the reports from this study (e.g., [40]), Khan and colleagues exclusively reported on the camera-based technology of DAAD, where fifteen cameras were installed in public spaces [29]. No primary data related to implementation outcomes were reported, with author narratives generally limited.

#### 3.9.1. Acceptability

Khan and colleagues noted that there were potential ethical concerns about the use of cameras regarding privacy and surveillance [29]. However, the authors did not indicate if these views were expressed by people with dementia, family members, and/or care staff.

#### 3.9.2. Adoption

Not reported.

#### 3.9.3. Appropriateness

There appeared to be issues with the appropriateness of the camera-based device in the DAAD study [29], the first being that the adoption of statically mounted cameras in a single room meant that the camera was unable to detect agitation events outside it. Another potential issue with appropriateness is that the camera would lead to false positives if there were new stimulus events in the room:

‘The downsides are that any novel or unusual visual stimuli will be triggered as events of interest, such as large pieces of equipment moving in the scene. Any clinical system based on this technology would need to have a way to handle anomalous ‘‘alerts’’ to minimize disruption from false positives.’

#### 3.9.4. Feasibility

Not reported.

#### 3.9.5. Fidelity

Not reported.

#### 3.9.6. Implementation Costs

Ye and colleagues noted that there was an ‘unexpected cost’ to consider with installing cameras [40]. The DAAD study took place in a hospital setting, requiring cables to be fire-rated and installation requiring a containment tent to prevent contamination.

## 4. Discussion

The use of technology to detect agitation in people with dementia in real-time is an area that has grown over the past two decades. Despite preliminary evidence of validity, research has failed to provide compelling evidence that these technologies can be successfully implemented for use with people with dementia. While more established technologies such as wearables show promise, there are still potential issues to overcome before they can be widely implemented in clinical and care practice for people with dementia.

The acceptability of real-time agitation detection technologies appears to be good. There is a pattern that less invasive and less interaction with the technology results in fewer acceptability issues in people with dementia. In part, by adopting limited interactive technologies, it overcomes potential factors related to gerontechnology acceptance, such as usability and user-friendliness [41]. Wearables may therefore be less acceptable to people with dementia as they attempt to interact with the devices, yet there were no specific reports that people with dementia disliked or found them problematic. Privacy concerns are also often highlighted within the broader literature [42], making camera-based systems less palatable. However, concerns over privacy again do not appear to feature prominently in the dementia agitation detection literature, outside of a few researchers’ commentaries.

There is a notable gap in the current literature, where people with dementia have not been meaningfully included in the evaluation process. When it comes to acceptability, carers (or staff) often become the primary respondents to interviews and survey items, or researchers make inferences about acceptability based on compliance. Engagement with stakeholders during the development of technology is seen as essential for successful implementation [43], whilst strategies to meaningfully include people with dementia in technology development and evaluation have previously been provided [44,45].

Across technology types, there was evidence of missing data, potentially indicating fidelity issues. For multimodal systems, it was not always clearly reported why there were gaps in data, though connectivity is a recurring theme. For wearables, the reasons for missing data were more clearly defined, such as the battery running out and wireless connectivity issues. If such technologies are adopted for clinical or care purposes, perhaps to complement care and support, then there is the question about what an acceptable level of missing data would be.

The appropriateness and feasibility of the technology tended to surround the extent to which the technology was able to continuously monitor the person with dementia. Issues with connectivity were a common theme, which reflects findings within the broader gerontechnology literature [46,47]. The majority of studies implemented technologies in residential or hospital settings; arguably, these settings are more controlled, but there were still technological issues. Two-thirds of people with dementia live in the community [48], and the variability of what ‘living in the community’ looks like raises further implementation issues, as well as brings into question the validity of generalising data from hospital or care home settings. For example, technology might not be feasible for use in rural settings when there is a reliance on an internet connection [33]. Even in high-income countries such as the UK, there is an infrastructure gap between those who live rurally and those who do not when it comes to the internet [49]. Another key issue for home or room-based sensors, or systems that require continuous internet connections, is that they will not be of use to the many people with dementia who leave their home setting while they are out. Whilst recognising how dementia can be isolating, with reports that around 50% of those in residential care never go outside [50], and those living in the community only go outside once a week [51], there are many people with dementia still maintaining regular outdoor engagement, including leisure time physical activity (54.6%) [52].

There are notable gaps in implementation outcomes, such as adoption and implementation costs. The question about cost is of particular concern, as these technologies may come at a high initial cost or even ongoing subscriptions, and these need to be considered in relation to the health benefit and other competing priorities [53]. There is also a general absence of primary data related to implementation outcomes. It is unclear to what extent the findings are generalisable, not least because sample sizes tended to be small (median = 10), but also because studies did not adequately describe their sample. For example, technologies that can be implemented in people with mild dementia with minimal agitation may not work in those with severe dementia and greater agitation. Public health intervention frameworks such as RE-AIM [54] emphasise reach and adoption as two key elements in successfully developing an intervention. Adopting such frameworks from the outset could assist in planning future real-time agitation detection technologies for people with dementia.

It was notable that the co-design of technology, or even the role of people with dementia in the technology’s development, was not commonly reported, which reflects findings from a previous review [16]. This broadly reflects the involvement of people with dementia in studies developing supportive technologies. One systematic review found that approximately half of the studies involved people with dementia in the generative phase, and only a small minority involved people with dementia in the pre-design phase [45]. There is a growing literature highlighting the value of co-design, in particular in ensuring that technologies meet the needs of people with dementia and are acceptable and usable [55,56].

### 4.1. Limitations

Studies in this review were only included if the technology was designed to or intended to detect agitation in real-time. This was to ensure that we captured technology with the intention of clinical or support purposes (e.g., to notify care staff when someone became agitated) and excluded a larger literature of devices that were exclusively collecting data for research purposes. As highlighted previously, the purpose of the technology may influence implementation outcomes, such as the perceived acceptability. However, implementation outcomes might be considered less critical if they have no bearing on the quality of care received.

The use of the critical appraisal tool sought to contextualise the included studies, though the QUADS tool used is not specifically designed to appraise implementation research. As such, the scores represent general study quality, rather than appraising the quality of the implementation research. Such an approach is probably appropriate, as the majority of studies (74%) did not refer to implementation outcomes as aims for the study. We were able to identify one critical appraisal tool for implementation research [57], though this tool has not yet been validated.

Only 4 (21%) of the studies were linked to an intervention, thus limiting the extent to which we can discuss their implementation as a continuation of the detection. In the limited literature available, carers being notified about agitation events may be acceptable [31], even if there are false positives [37]. We synthesised data based on technology type [10] and implementation outcome [11]. We note that during the synthesis process, it was not always clear what type of technology or type of implementation outcome was being reported. Consensus between researchers minimised potential sources of bias. Ultimately, categorising the studies in this way may simplify the complexity of the technology and outcomes.

### 4.2. Recommendations

Based on the current state of the literature, future research should achieve the following:

1. Clearly define implementation outcomes and consider how they will be measured.
2. Ensure that the voices of key stakeholders are included and reported during the development of the technologies.
3. Ensure that people with dementia are meaningfully included when ascertaining acceptability, appropriateness, and other implementation outcomes.
4. Clearly describe the sample to better understand the generalisability for people in different stages of dementia and different levels and types of agitation.
5. Reflect on the extent to which the technology is appropriate and feasible for use in people with dementia living in the community.
6. Ascertain what level of missing data is considered appropriate to enable the technology to be used for healthcare purposes.

## 5. Conclusions

While technology for real-time agitation detection in people with dementia shows promise, particularly for wearables, there are significant challenges to overcome before widespread clinical implementation. Although acceptability is generally good, especially for less invasive technologies, there is a need for more direct involvement of people with dementia in their design and evaluation process. Implementation outcomes have not been a key focus of research in real-time agitation detection technology for people with dementia. There are general technological issues that limit implementation. In summary, there are clear gaps in our knowledge about whether and how such technologies meet the needs of people with dementia, carers and staff.

## Figures and Tables

**Table 1:** Early-stage implementation outcomes.

Implementation Outcome	Definition
Acceptability	The perception among implementation stakeholders that a given treatment, service, practice, or innovation is agreeable, palatable, or satisfactory.
Adoption	The intention, initial decision, or action to try or employ and innovation or evidence-based practice (may also be referred to as ‘uptake’).
Appropriateness	The perceived fit, relevance, or compatibility of the innovation or evidence-based practice for a given practice setting, provider, or consumer; and/or perceived fit of the innovation to address a particular issue or problem (overlapping with acceptability).
Feasibility	The extent to which a new treatment, or an innovation, can be successfully used or carried out within a given agency or setting (invoked retrospectively as a potential explanation of an initiative’s success or failure, as reflected in poor recruitment/retention/participation rates).
Fidelity	The degree to which an intervention was implemented as it was prescribed in the original protocol or as it was intended by the programme developers.
Implementation cost	The cost impact of an implementation effort.

Adapted from Proctor et al., 2011 [11].

**Table 2:** Descriptive data surrounding the characteristics of technology used and its deployment.

Author, Year, Country	Part of Study	Description of Technology/Agitation Sensing Modalities	Sample Size (*n*)	Planned (or Maximum) Detection Duration Continuous/Time Specific (No. Days/Hours Deployed)	Detection Linked to Intervention	Setting
Multimodal						
Alam et al. (2017) USA [30]	BESI	Wearables (smart-watches); other ambient sensors (in-home environmental sensors)	2	Continuous (30 days)	No	Home/community
Anderson et al. (2021) USA [31]	BESI and CANIS	Wearables (smart-watches); other Ambient sensors (in-home environmental sensors)	10	Continuous (60 days)	Yes	Home/community
Au-Yeung et al. (2020) USA [32]	MODERATE	Wearable (Actiwatch); other ambient sensors (in-home environmental sensors and bed pressure mats)	1	Continuous (138 days)	No	Residential care home
Bankole et al. (2020) USA [33]	BESI	Wearables (smart-watches); other ambient sensors (in-home environmental sensors)	12	Continuous (30 days)	No	Home/community
Badawi et al. (2023) Canada [34]	DAAD	Wearables (smartwatch); camera-based (computer vision)	17	Continuous (60 days)	No	Hospital (inc. long-term and/or care unit)
Davidoff et al. (2022) Belgium [35]	-	Ambient sensors (in-home environmental sensors) and wearables (bracelet, belt, and button)	1	Time-specific (1 day)	No	Hospital (inc. long-term and/or care unit)
Gong et al. (2015) USA [36]	-	Wearable (bracelet); other ambient sensors (bed pads and microphone)	12	Time specific (>38 days)	No	Home/community
Homdee et al. (2019) USA [37]	BESI	Wearables (smartwatches), other ambient sensors (in-home environmental sensors)	17	Continuous (60 days)	Yes	Home/community
Khan et al. (2023) Canada [22]	DAAD	Wearables (smartwatch); camera-based (computer vision)	20	Time-specific (60 days)	No	Hospital (inc. long-term and/or care unit)
Rose et al. (2015) USA [38]	-	Wearables (watch-like device), other ambient sensor (acoustic sensor and sensor pads)	Not reported	Time-specific (>7 days)	No	Home/community
Spasojevic et al. (2021) Canada [39]	DAAD	Wearables (smartwatch); camera-based (computer vision)	17	Continuous (60 days)	No	Hospital (inc. specialised care unit)
Ye et al. (2019) Canada [40]	DAAD	Wearables (smartwatch); camera-based (computer vision); ambient sensors (sensor pad)	11	Continuous (60 days)	Yes	Hospital (inc. specialised care unit)
Wearables						
Alam et al. (2019) USA [23]	BESI	Wearables (smartwatch),	10	Continuous (30 days)	No	Home/community
Bankole et al. (2011) USA [24]	-	Wearables (wrist, ankle, and waist)	6	Time specific (3 h)	No	Residential care home
Nesbitt et al. (2018) USA [25]	-	Wearables (watch and phone)	8	Time-specific (1 day)	No	Residential care home
Teipel et al. (2017) Germany [26]	InsideDem	Wearables (wrist and ankle)	17	Continuous (>28 days)	no	Residential care home
Other ambient sensor						
Banerjee et al. (2004) France [27]	-	Passive infrared sensors	3	Time specific (>63 days)	No	Hospital (inc. long-term and/or care unit)
Vahia et al. (2020) USA [28]	-	Passive infrared sensors	1	Continuous (70 days)	No	Residential care home
Camera based (computer vision)						
Khan et al. (2022) Canada [29]	DAAD	Cameras installed in public spaces	1	Time-specific (60 days)	No	Hospital (inc. long-term and/or care unit)

**Table 3:** Reported implementation outcomes.

Author, Year	Implementation Outcomes Included in Aims of Report?	Acceptability	Adoption	Appropriateness	Feasibility	Fidelity	Implementation Cost
Multimodal							
Alam et al. (2017) [30]	Yes			S		P	
Anderson et al. (2021) [31]	Yes	P; S		P			
Au-Yeung et al. (2020) [32]	No	S				P	
Bankole et al. (2020) [33]	No				S	P	
Badawi et al. (2023) [34]	No	S			S		
Davidoff et al. (2022) [35]	No				S	S	
Gong et al. (2015) [36]	Yes	P				S	
Homdee et al. (2019) [37]	No	P; S			P	S	
Khan et al. (2023) [22]	No			S			
Rose et al. (2015) [38]	Yes	S					
Spasojevic et al. (2021) [39]	No					S	
Ye et al. (2019) [40]	Yes				S		S
Wearables							
Alam et al. (2019) [23]	No	S					
Bankole et al. (2011) [24]	No	P; S				P	
Nesbitt et al. (2018) [25]	No			S			
Teipel et al. (2017) [26]	Yes	P; S		P	P	P	
Other Ambient Sensor							
Banerjee et al. (2004) [27]	No			S			
Vahia et al. (2020) [28]	No	S				P	
Camera Based (Computer Vision)							
Khan et al. (2022) [29]	No			S			

P = primary data; S = secondary data (i.e., researcher commentary).

## Data Availability

No new data were created or analysed in this study. Data sharing is not applicable to this article.

## Abbreviations

The following abbreviations are used in this manuscript:

QUADS	Quality Assessment with Diverse Studies.
DAAD	Detect Agitation and Aggression in Dementia.
BESI	The Behavioral and Environmental Sensing and Intervention.
CANIS	Caregiver-Personalized Automated Non-Pharmacological Intervention System.
TRL	Technology Readiness Level.
NIHR	National Institute for Health Research.

## Appendix A

**Section and Topic**	**Item #**	**Checklist Item**	**Location Where Item Is Reported ***
**TITLE**			
Title	1	Identify the report as a systematic review.	Title/Pg1
**ABSTRACT**			
Abstract	2	See the PRISMA 2020 for Abstracts checklist.	Pg 1
**INTRODUCTION**			
Rationale	3	Describe the rationale for the review in the context of existing knowledge.	Pg 1–2
Objectives	4	Provide an explicit statement of the objective(s) or question(s) the review addresses.	Pg 3
**METHODS**			
Eligibility criteria	5	Specify the inclusion and exclusion criteria for the review and how studies were grouped for the syntheses.	Pg 4
Information sources	6	Specify all databases, registers, websites, organisations, reference lists and other sources searched or consulted to identify studies. Specify the date when each source was last searched or consulted.	Pg 4
Search strategy	7	Present the full search strategies for all databases, registers and websites, including any filters and limits used.	Page 3–4, Appendix B
Selection process	8	Specify the methods used to decide whether a study met the inclusion criteria of the review, including how many reviewers screened each record and each report retrieved, whether they worked independently, and if applicable, details of automation tools used in the process.	Page 4
Data collection process	9	Specify the methods used to collect data from reports, including how many reviewers collected data from each report, whether they worked independently, any processes for obtaining or confirming data from study investigators, and if applicable, details of automation tools used in the process.	Page 4–5
Data items	10a	List and define all outcomes for which data were sought. Specify whether all results that were compatible with each outcome domain in each study were sought (e.g., for all measures, time points, analyses), and if not, the methods used to decide which results to collect.	Pages 4–5
	10b	List and define all other variables for which data were sought (e.g., participant and intervention characteristics, funding sources). Describe any assumptions made about any missing or unclear information.	Pages 4–5
Study risk of bias assessment	11	Specify the methods used to assess risk of bias in the included studies, including details of the tool(s) used, how many reviewers assessed each study and whether they worked independently, and if applicable, details of automation tools used in the process.	Page 5
Effect measures	12	Specify for each outcome the effect measure(s) (e.g., risk ratio, mean difference) used in the synthesis or presentation of results.	N/A
Synthesis methods	13a	Describe the processes used to decide which studies were eligible for each synthesis (e.g., tabulating the study intervention characteristics and comparing against the planned groups for each synthesis (item #5)).	Page 6
	13b	Describe any methods required to prepare the data for presentation or synthesis, such as handling of missing summary statistics, or data conversions.	N/A
	13c	Describe any methods used to tabulate or visually display results of individual studies and syntheses.	N/A
	13d	Describe any methods used to synthesize results and provide a rationale for the choice(s). If meta-analysis was performed, describe the model(s), method(s) to identify the presence and extent of statistical heterogeneity, and software package(s) used.	Page 6
	13e	Describe any methods used to explore possible causes of heterogeneity among study results (e.g., subgroup analysis, meta-regression).	N/A
	13f	Describe any sensitivity analyses conducted to assess robustness of the synthesized results.	N/A
Reporting bias assessment	14	Describe any methods used to assess risk of bias due to missing results in a synthesis (arising from reporting biases).	Pg 6
Certainty assessment	15	Describe any methods used to assess certainty (or confidence) in the body of evidence for an outcome.	Page 6
**RESULTS**			
Study selection	16a	Describe the results of the search and selection process, from the number of records identified in the search to the number of studies included in the review, ideally using a flow diagram.	Page 7
	16b	Cite studies that might appear to meet the inclusion criteria, but which were excluded, and explain why they were excluded.	-
Study characteristics	17	Cite each included study and present its characteristics.	Table 1
Risk of bias in studies	18	Present assessments of risk of bias for each included study.	Appendix C
Results of individual studies	19	For all outcomes, present, for each study: (a) summary statistics for each group (where appropriate) and (b) an effect estimate and its precision (e.g., confidence/credible interval), ideally using structured tables or plots.	N/A
Results of syntheses	20a	For each synthesis, briefly summarise the characteristics and risk of bias among contributing studies.	Page 8
	20b	Present results of all statistical syntheses conducted. If meta-analysis was done, present for each the summary estimate and its precision (e.g., confidence/credible interval) and measures of statistical heterogeneity. If comparing groups, describe the direction of the effect.	N/A
	20c	Present results of all investigations of possible causes of heterogeneity among study results.	N/A
	20d	Present results of all sensitivity analyses conducted to assess the robustness of the synthesized results.	N/A
Reporting biases	21	Present assessments of risk of bias due to missing results (arising from reporting biases) for each synthesis assessed.	N/A
Certainty of evidence	22	Present assessments of certainty (or confidence) in the body of evidence for each outcome assessed.	N/A
**DISCUSSION**			
Discussion	23a	Provide a general interpretation of the results in the context of other evidence.	Pages 17–18
	23b	Discuss any limitations of the evidence included in the review.	Pages 18–19
	23c	Discuss any limitations of the review processes used.	Pages 18–19
	23d	Discuss implications of the results for practice, policy, and future research.	Page 19
**OTHER INFORMATION**			
Registration and protocol	24a	Provide registration information for the review, including register name and registration number, or state that the review was not registered.	Page 3
	24b	Indicate where the review protocol can be accessed, or state that a protocol was not prepared.	Page 3
	24c	Describe and explain any amendments to information provided at registration or in the protocol.	N/A
Support	25	Describe sources of financial or non-financial support for the review, and the role of the funders or sponsors in the review.	Page 20
Competing interests	26	Declare any competing interests of review authors.	Page 20
Availability of data, code and other materials	27	Report which of the following are publicly available and where they can be found: template data collection forms; data extracted from included studies; data used for all analyses; analytic code; any other materials used in the review.	N/A
	* In submitted manuscript format. From: Page MJ, McKenzie JE, Bossuyt PM, Boutron I, Hoffmann TC, Mulrow CD, et al. The PRISMA 2020 statement: an updated guideline for reporting systematic reviews. BMJ 2021;372:n71. doi: 10.1136/bmj.n71 [19]. This work is licensed under CC BY 4.0.		

## Appendix B

TITLE-ABS-KEY (detect* OR monitor* OR automated OR real-time) AND TITLE-ABS-KEY (technology OR monitoring OR physiologic* OR ‘signal processing’ OR computer assisted’ OR accelerometry OR actigraph OR ‘vital signs’ OR ‘heart rate’ OR wearable OR sensor OR ‘machine learning’ OR ‘artificial intelligence’ OR ‘electrocardiography OR wrist OR worn OR body OR video OR recording OR camera OR ‘pressure mat’) AND TITLE-ABS-KEY (‘Behavioural and psychological symptoms’ OR BPSD OR agitation OR aggression OR ‘motor behaviours’ OR neuropsychiatric) AND TITLE-ABS-KEY (dementia OR Alzheimer* OR resident* OR neurocognitive).

## Appendix C

**Table A1:** QUADS score for included studies.

First Author	Year	Citation	Research Theory/ Concept	Research Aim	Research Setting and Population	Study Design	Sampling Method	Data Collection Rationale	Data Collection Format	Data Collection Procedure	Recruitment Data	Analytic Method Justification	Analysis Method	Stakeholder Consideration	Strengths and Limitations	Total	%
Homdee	2019	[37]	2	1	2	1	0	1	1	1	0	0	0	0	1	10	26
Tiepel	2017	[26]	3	3	3	2	2	3	3	2	0	0	3	2	3	29	74
Au-Yeung	2020	[32]	2	3	3	3	2	3	3	3	0	3	3	1	2	31	79
Banerjee	2004	[27]	1	1	1	1	1	1	1	1	0	0	0	1	1	10	26
Spasojevic	2021	[39]	3	3	2	3	3	3	3	3	1	2	3	1	3	33	85
Gong	2015	[36]	1	2	1	2	0	3	3	3	0	3	3	1	2	24	62
Bankole	2011	[24]	3	2	2	3	2	3	3	3	0	3	3	1	3	31	79
Ye	2019	[40]	3	3	3	3	2	3	3	3	1	3	3	2	3	35	90
Vahia	2020	[28]	2	2	3	2	2	2	3	2	0	2	2	0	2	24	62
Nesbitt	2018	[25]	3	3	2	2	2	2	2	2	1	3	3	1	2	28	72
Badawi	2023	[34]	3	2	2	3	2	1	3	2	1	3	3	1	2	28	72
Rose	2015	[38]	3	2	2	3	3	3	3	2	1	0	0	2	2	26	67
Alam	2019	[23]	2	1	2	2	1	1	1	2	1	3	3	1	2	22	56
Khan	2022	[29]	2	2	3	2	3	3	3	3	1	3	2	2	3	32	82
Bankole	2020	[33]	3	3	2	3	2	3	3	3	2	3	3	1	3	34	87
Alam	2017	[30]	2	2	1	2	0	3	2	3	1	1	1	1	2	21	54
Anderson	2021	[31]	2	3	3	3	3	3	3	3	3	2	3	3	2	36	92
Davidoff	2022	[35]	3	3	3	3	2	3	3	3	2	3	3	1	3	35	90
Khan	2023	[22]	3	2	3	3	3	3	3	3	1	2	2	1	3	32	82

Score: 0 = not described, to 3 = appropriate and explicitly described.
